# Giant intrinsic spin Hall effect in W_3_Ta and other A15 superconductors

**DOI:** 10.1126/sciadv.aav8575

**Published:** 2019-04-05

**Authors:** E. Derunova, Y. Sun, C. Felser, S. S. P. Parkin, B. Yan, M. N. Ali

**Affiliations:** 1 Max Plank Institute for Microstructure Physics, Halle, Germany.; 2 Max Planck Institute for Chemical Physics of Solids, Dresden, Germany.; 3 Department of Condensed Matter Physics, Weizmann Institute of Science, Rehovot, Israel.

## Abstract

The spin Hall effect (SHE) is the conversion of charge current to spin current, and nonmagnetic metals with large SHEs are extremely sought after for spintronic applications, but their rarity has stifled widespread use. Here, we predict and explain the large intrinsic SHE in β-W and the A15 family of superconductors: W_3_Ta, Ta_3_Sb, and Cr_3_Ir having spin Hall conductivities (SHCs) of −2250, −1400, and 1210 $\frac{\hbar }{e}(S/\text{cm})$, respectively. Combining concepts from topological physics with the dependence of the SHE on the spin Berry curvature (SBC) of the electronic bands, we propose a simple strategy to rapidly search for materials with large intrinsic SHEs based on the following ideas: High symmetry combined with heavy atoms gives rise to multiple Dirac-like crossings in the electronic structure; without sufficient symmetry protection, these crossings gap due to spin-orbit coupling; and gapped crossings create large SBC.

## Teaser

We identify electronic structure descriptors for the spin Hall effect and propose a design strategy and several new SHE materials.

## INTRODUCTION

The spin Hall effect (SHE) has become an important topic in recent years not only from a fundamental physics aspect but also in regard to near-future technological application. This is due to a combination of Moore’s law limits on traditional Si-based devices and the concurrent rise of spintronics, creating logic and storage devices based on manipulating both spin and current (*1*). Spintronics has become the next evolution in computing technology and is already seeing commercial technological adoption. In particular, the study of spin transfer phenomena, where the magnetization of a ferromagnet is manipulated through the transfer of spin angular momentum from a spin current, is considered a promising direction (*2*, *3*). However, the creation of large spin currents, ideally at low power, at room temperature, and using low-cost materials amenable to facile device fabrication, remains a serious challenge. The three major routes to achieving these criteria are (i) using heterostructures of ferromagnetic metals and nonmagnetic semiconductors, (ii) using ferromagnetic semiconductors, or (iii) using nonmagnetic metals and the SHE (*2*).The direct conversion of charge current to spin current via the SHE is highly appealing for spin orbit torque and spin transfer torque devices, both of which are being investigated for magnetic random access memory purposes (*4*, *5*). However, the magnitude of the SHE in nonmagnetic metals has been low; simple 4d and 5d elements have spin Hall conductivities (SHCs) calculated to be just a few hundred $\frac{\hbar }{e}(S/\text{cm})$ with the notable exceptions of Ta [body-centered cubic (BCC)], W (BCC), and Pt [face-centered cubic (FCC)] (*6*, *7*). Recently, Weyl semimetals have been explored as SHE materials due to topologically derived spin Berry curvature (SBC) with intrinsic SHC magnitudes calculated to be 700 to 800 $\frac{\hbar }{e}(S/\text{cm})$ (*8*).

The ratio of generated spin current to charge current is defined as the spin Hall angle (SHA), and larger SHAs are desired for technological applications. Topological insulators (TIs) such as Bi_2_Se_3_ have been heavily investigated in recent years, and various studies have found SHAs widely ranging from 0.01 to 425; hence, their effectiveness as spin Hall materials is debated. Large-scale fabrication methods for these materials remain challenging; however, recent progress has been made (e.g., Bi_*x*_Se_1−*x*_ and BiSb) (*9*–*14*). In addition, large SHEs for spin orbit torque switching have recently been shown in some TIs (*15*–*17*). Pt/doped Pt, β-Ta, and recently β-W/doped β-W are the only known sputterable materials to host very large SHAs at room temperature (*18*–*22*). Recent first-principles studies on β-W have predicted it to have a larger SHE than α-W and that W_1−*x*_Ta_*x*_ alloys can have even higher SHEs (*23*). Here, we explain why β-W has a giant SHE, based on its symmetry and electronic structure, and predict several more sputterable, low-cost, and giant SHE materials in the A15 family of superconductors. We also propose a simple and rapid search strategy for finding materials with large SHEs that represents the first application of concepts from topological physics in industrial technology and, with tuning of extrinsic parameters, will markedly affect today’s commercially relevant spin-based technologies.

In the normal SHE, passing a current through a spin Hall material generates an orthogonal spin current, which is polarized perpendicularly to both the charge and spin current directions. There are several mechanisms by which the SHE can be achieved, and they can be generally grouped into two categories: extrinsic mechanisms and intrinsic mechanisms. Extrinsic mechanisms refer to the methods by which a spin acquires a transverse velocity from the scattering of electrons due to spin-orbit coupling (SOC) (*24*, *25*). Impurity and defect scattering are the most common causes. In the intrinsic mechanism, on the other hand, the spin current is created in between scattering events rather than during them (*24*, *26*).

**Fig. 1:**
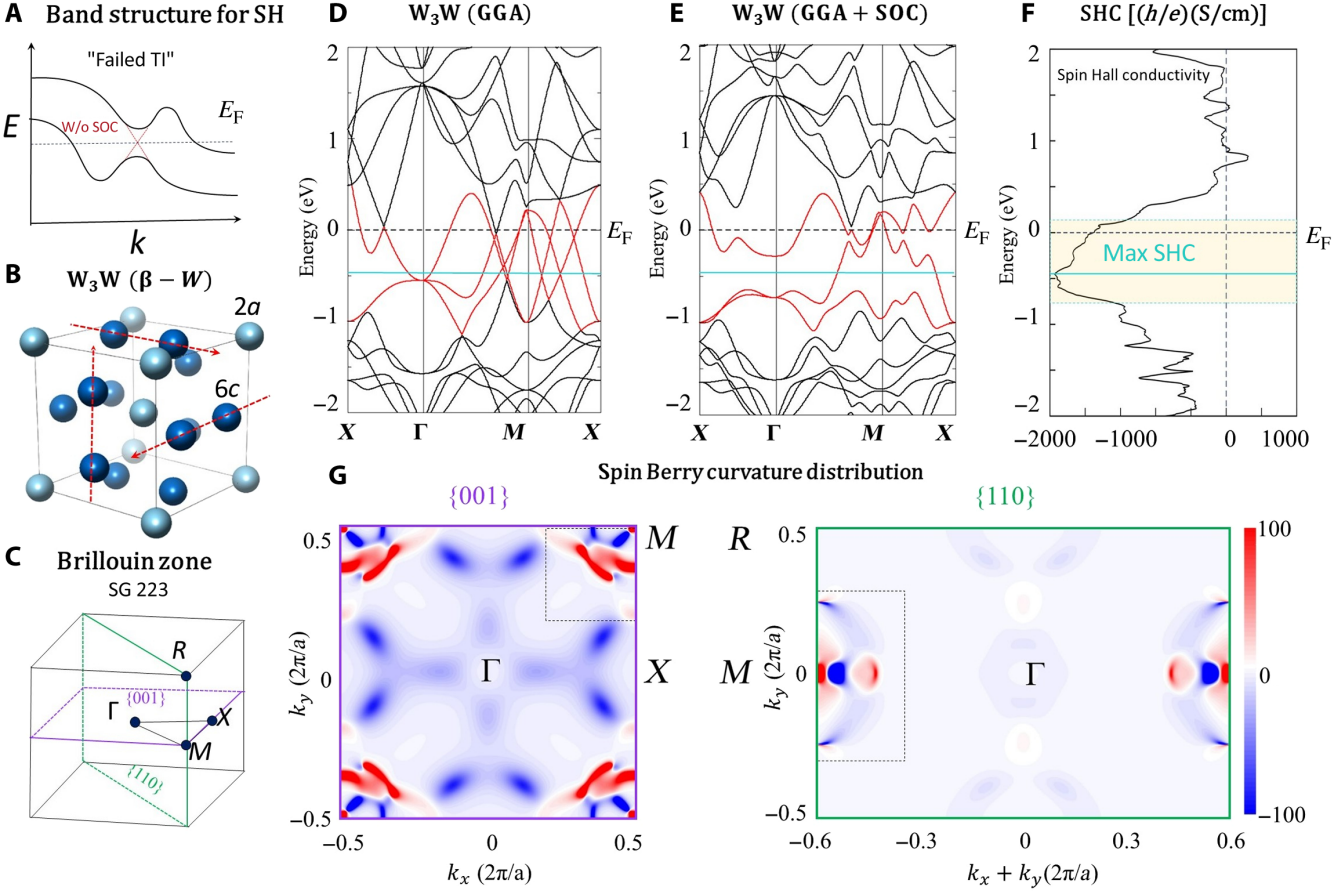
Crystal and electronic structures of β-W as well as the SHC and SBC distribution. (**A**) Schematic of the “failed TI” band structure that is good for spin Hall purposes. (**B**) Crystal structure of W_3_W, also known as β-W, prototype of the A15 family. Light and dark blue balls represent the two different crystallographic sites, and red arrows show the orthogonal infinite chains formed by atoms at the 6*c* site. (**C**) Brillouin zone (BZ) for A15 family (SG 223). (**D** and **E**) Electronic structures of W_3_W without and with SOC, respectively. Dirac crossings are visible (without SOC) along the Γ-*X*-*M* lines, both at and below *E*_F_. (**F**) SHC versus energy plot of W_3_W. The yellow area marks the region of high SHC, and the light blue line marks the maximum SHC. (**G**) SBC distribution in the 001 and 110 planes of the BZ of W_3_W, where red and blue areas represent positive and negative regions, respectively.

The SHC can be calculated using a linear response approach in the Streda-Kubo formalism (*27*, *28*). In this case, the SHC is split into two parts, σ^*I*^ and σ^*II*^ (*6*, *29*)

$$\sigma_{xy}^{zI}=\frac{1}{2\pi N}\sum_{k}Tr{\left[{\hat{J}}_{x}^{S}{\hat{G}}^{R}{\hat{J}}_{y}^{C}{\hat{G}}^{A}\right]}_{\omega =0}$$ (1)

$$\sigma_{xy}^{zII}=\frac{-1}{4\pi N}\sum_{k}\int_{-\infty }^{0}Tr[{\hat{J}}_{x}^{S}\frac{\partial {\hat{G}}^{R}}{\partial \omega }{\hat{J}}_{y}^{C}{\hat{G}}^{R}-{\hat{J}}_{x}^{S}\partial {\hat{G}}^{R}{\hat{J}}_{y}^{C}\frac{{\hat{G}}^{R}}{\partial \omega }-<R↔A>]$$ (2)

where ${\hat{J}}_{x}^{S}$ is an *s*_*z*_-spin current operator, ${\hat{J}}_{y}^{C}$ is a charge current operator, and ${\hat{G}}^{R}\text{and}\;{\hat{G}}^{A}$ are retarded and advanced Green functions. In the presence of Dirac and quadratic band crossings (like in Luttinger semimetals), $\sigma_{xy}^{zI}=0$ and the main contribution to the SHC comes from $\sigma_{xy}^{zII}$. When the quasiparticle damping rate is equal to 0 (i.e., pure intrinsic regime), $\sigma_{xy}^{zII}$ is reduced to the following expression

$$\sigma_{xy}^{zIIb}=\frac{1}{N}\sum_{k,l}f(E_{k}^{l})\Omega^{l}(k)$$ (3)

where $f(E_{k}^{l})$ is a Fermi distribution function and Ω^*l*^(*k*) is referred to as the SBC, part of a broader concept arising from the *k* dependence of the wave function, ψ_*n*_(*k*) (*30*). ψ_*n*_(*k*) is heavily influenced by the crystalline symmetries that drive orbital hybridization and thus directly influence the Berry curvature and SBC. Bands that create a crossing but then also form a hybridization gap with the inclusion of SOC (also known as an anticrossing), as shown schematically in Fig. 1A, will give rise to a large SBC. This is because the SBC is opposite for bands on either side of the hybridization gap, but when *E*_F_ lies inside the gap, the oppositely signed contributions are not compensated, as can be seen in β-W’s SBC distribution (Fig. 1G) (*24*). Because the intrinsic SHE is directly proportional to the integration of the SBC of each occupied band, gapped crossings generate large SHEs. If one is aiming for a maximized SHE, a reasonable starting point is a system with the presence of these gapped crossings. This is the well-known cause of the large intrinsic SHE in Pt, where the Fermi level lies inside gapped crossings near the *L* and *X* points in the BZ (see fig. S1) (*6*, *26*, *29*, *31*). When the Berry curvature of all occupied bands is integrated over the entire BZ, Pt naturally has a peak in its SHC versus energy plot at *E*_F_. The mixing of orbital character caused by SOC-driven hybridization is also visible in fig. S1. The magnitude of the SHE is inversely proportional to the size of the SOC-induced band gap; too large of a gap results in a low SHE.

To create a large SHA for use in spintronics, large SHEs are desired, and both extrinsic and intrinsic effects can contribute substantially to the overall magnitude of the generated spin current (*24*). Ideally, maximizing the SHE will be a combined effort of first picking a material with a large intrinsic effect and then maximizing extrinsic effects through interfaces, doping, defect control, etc. Few materials are known with large intrinsic effects, especially materials that are amenable to large-scale thin-film fabrication through sputtering. However, a simple strategy to search for or design new SHE materials is to maximize intrinsic SHE by maximizing Berry curvature by having *E*_F_ inside as many small-gapped crossings as possible.

The recent search for TIs, Dirac, Weyl, and nodal line semimetals has relied on an understanding of the effects of crystal symmetry on the electronic structure. The symmetry of a crystal structure and the orbitals making up the electronic states when bands are crossed determine which topological state is realized when SOC is considered. Electronic states must be mutually orthogonal to not hybridize with each other and open a gap. The various symmetry operations can all create degeneracies in the band structure at specific points or along particular directions (*32*, *33*). Some of these symmetries, or combinations of them, can create and protect a degeneracy from being gapped by the inclusion of SOC. For example, the *C*_2*v*_ point group (spinless case) has four irreducible representations, but the *C*_2*v*_ double group (spinful case) has only one irreducible representation. Bands with the same irreducible representation (meaning not orthogonal) hybridize, so spinful band crossings with only *C*_2*v*_ symmetry gap in the presence of SOC (*34*). On the other hand, the presence of a glide mirror, *G*, which has a fraction of a primitive unit vector translation operation, *t*, results in *G*^2^ = *e*^−*i***k**⋅**t**^ for Bloch states, making the glide eigenvalues equal to ±*e*^−*i***k**⋅**t**^ (±*ie*^−*i***k**⋅**t**^) for spinless (spinful) systems. Therefore, regardless of SOC, the glide mirror, which is a nonsymmorphic symmetry operation, yields two distinct eigenvalues and the bands are protected from hybridizing and opening up a gap (*35*, *36*).

If one analyzes the crystallographic symmetry, it is possible to determine whether a system must have unprotected and protected crossings and even along which *k*-paths they lie. Recently, there has been an intense effort to use symmetry and group theory to create a complete analysis of all topological classes possible in the 230 space groups (*37*, *38*). It turns out that protected crossings are not rare, and unprotected crossings are commonplace. However, unlike the usual aim of materials scientists working in the topological field, where the goal has been typically to put protected crossings at the Fermi level, here, the materials scientist’s goal, for spin Hall [and anomalous Hall (AHE) in the case of time-reversal symmetry breaking] purposes, is to put unprotected crossings at the Fermi level. Good SHE and AHE materials will have enough symmetry to demand crossings, but not the right symmetries to protect those crossings against SOC, at *E*_F_. This means that many of the materials that were once investigated as potential Dirac/Weyl semimetals but had SOC-driven gap openings, or were investigated as potential TIs but had additional metallic bands, are worth reexamining for their SHE and AHE. Figure 1A shows a simple schematic of a “failed” TI band structure, which, like Pt, results in a peak in the intrinsic SHC.

## RESULTS

β-W, particularly when doped with small amounts of oxygen (few percent), is also known to have an enormous SHA as large as −0.45, which has been successfully used in spin transfer torque devices (*18*, *39*). To the best of our knowledge, no explanation has been given for its large SHC and correspondingly large SHA. However, it can be understood with the concepts outlined above; β-W has a large intrinsic SHE due to several unprotected crossings near the Fermi level, resulting in a net large SBC. Figure 1B shows the crystal structure of β-W, also known as W_3_W, the prototype of the A15 structure type (*A*_3_*X*, space group 223, *Pm*-3*n*) that is famous for hosting high critical current superconductors such as Nb_3_Sn, which are still the most widely used superconductors in technological applications today (*40*). The structure has two distinct crystallographic sites (6*c* and 2*a*) and can be thought of as a BCC lattice made by the X atom with two A atoms in each face of the cube. The BZ for this system is shown in Fig. 1C, with several key high-symmetry points listed. The electronic structures of β-W, both without and with SOC, are shown in Fig. 1 (D and E, respectively). Bands that, without SOC, create several Dirac crossings very close to the Fermi level along the Γ-*X*-*M* lines, as well as below *E*_F_, are shown in red. These crossings are created by *C*_2_ rotation and inversion symmetries: For example, the crossing along Γ-*X* at *E*_F_ is protected by *C*_2_ rotations along the (010) and (001) axes coupled with an inversion operation. However, the symmetries protecting these crossings all belong to the *C*_2*v*_ point group, which, as described earlier, can create degeneracies without SOC but gap due to SOC. As expected, with the inclusion of SOC in Fig. 1E, these bands gap out, and the Fermi level lies almost within those gaps. Correspondingly, a broad peak in the SHC versus energy calculation (Fig. 1F) straddles the energies where the gapped crossings lie. This creates “hotspots” of SBC, indicated by the intense red and blue areas in Fig. 1G, in the BZ precisely where the crossings were. The maximum SHC actually lies approximately 0.5 eV below *E*_F_ at the intersection of several more gapped crossings, and if *E*_F_ were lowered by hole doping, without notably altering the band dispersion characteristics, it is expected that the SHC could be maximized.

**Fig. 2:**
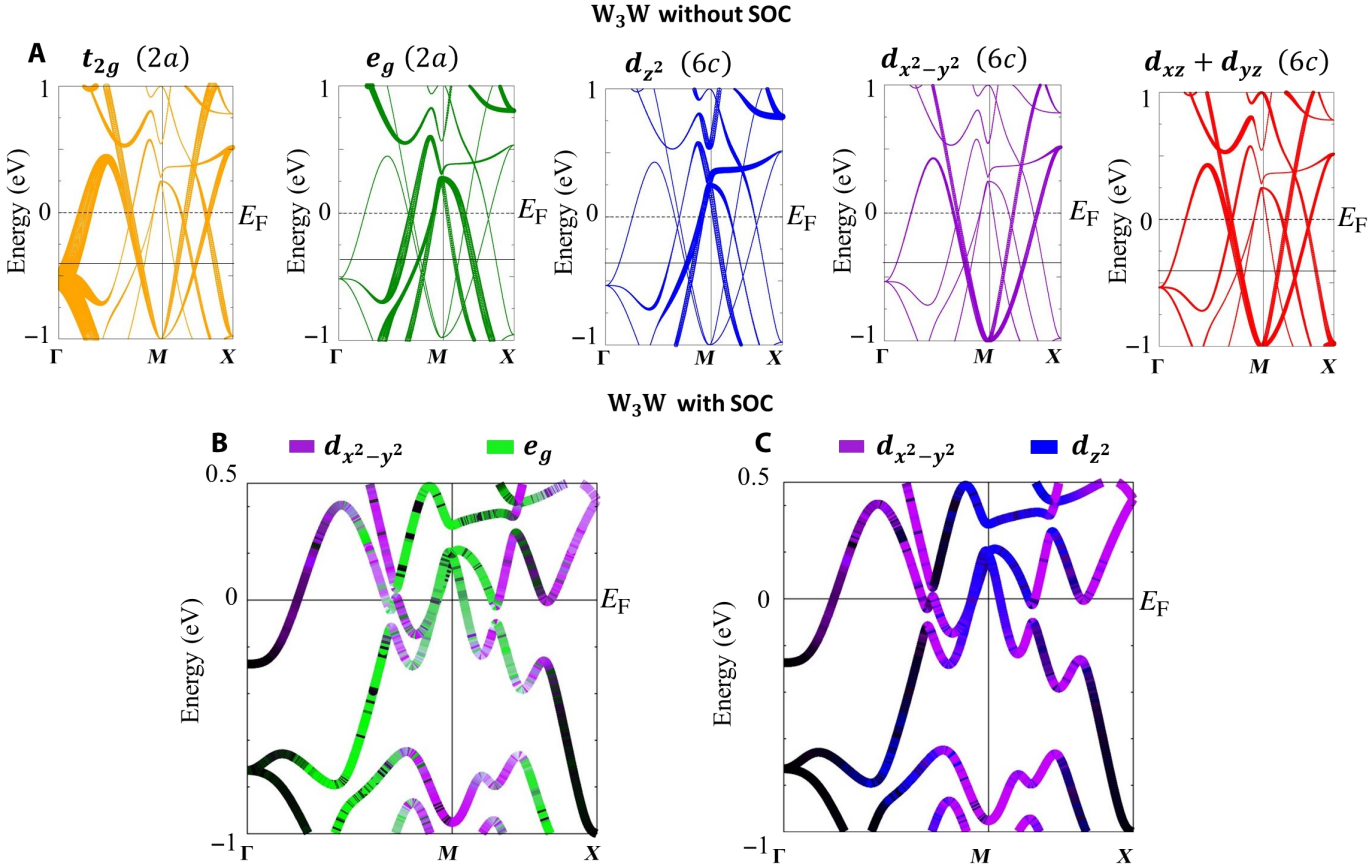
Orbital character contributions to the electronic bands of W_3_W. (**A**) Electronic structure of W_3_W near the Dirac crossings decomposed into the various orbital and crystallographic site (2*a* and 6*c*) contributions. The thickness of the bands indicates the extent of the orbital or orbital group contribution to a band. By symmetry, the 2*a* site’s orbitals group into the *t*_2*g*_ and *e*_*g*_ sets. The 6*c* site has lower degeneracy. *d*_*xy*_ is not shown as it does contribute to the relevant bands. (**B** and **C**) Electronic structures with SOC included, illustrating the orbital hybridization driven by SOC, opening gaps and generating SBC.

Figure 2A shows the electronic structure without SOC of β-W broken apart by the crystallographic site and orbital contributions to the bands. The 2*a* site contributes almost exclusively to the degeneracy at Γ via the *t*_2*g*_ orbitals, while the 6*c* site mixes with orbitals from the 2*a* site to create the crossings along Γ-*X*-*M* both near *E*_F_ and below it. Without SOC, β-W has highly dispersive linear bands and Dirac crossings akin to a Dirac semimetal. Figure 2 (B and C) illustrates the extent of the orbital hybrdization when SOC is included, which gaps the Dirac crossings. The changing color of the bands, particularly along the *M*-*X* direction below *E*_F_, indicates the change of character from $d_{x^{2}-y^{2}}$ to the *e*_g_ and $d_{z^{2}}$ orbitals, respectively. This is analogous to what occurs in Pt (fig. S1), where strongly hybridized bands gap its Dirac crossing and result in mixed orbital character and large SBC as well.

**Fig. 3:**
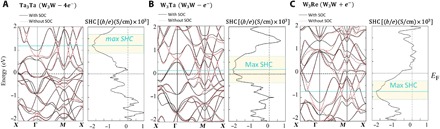
(**A** to **C**) Electronic structures of Ta_3_Ta, W_3_Re, and W_3_Ta, without and with SOC included, as well as their SHC versus energy plots.

The band structure of Ta_3_Ta, a hypothetical A15 version of Ta where both crystallographic sites are occupied by Ta, is shown in Fig. 3A. Because of the similarity of Ta and W, this can be thought of as β-W with four electrons removed, while Fig. 3 (B and C) shows the band structures for W_3_Ta and W_3_Re, respectively, also in the A15 structure type. However, in W_3_Re, the 2*a* site has been replaced with Re (adding one electron to β-W), while in W_3_Ta it was replaced with Ta (subtracting one electron from β-W). As can be seen from the band structures, the major features, including the Dirac crossings seen in β-W without SOC, are preserved because they are symmetry demanded. Interchanging Ta with W and Re does not fundamentally break the crystalline or band symmetries. In addition, Fig. 3 (A and B) implies that Ta/W site ordering is not critical to manifestation of the Dirac crossings and that even in a disordered thin film, as is expected from sputtered growth, the gapped crossings will persist. W_3_Ta has shifted the Dirac crossings nearly exactly to the Fermi level and has a maximum calculated SHC of 2250 $\frac{\hbar }{e}(S/\text{cm})$, one of the highest values for any known compound. Thin films of this material, with expected interfacial spin transparency and conductivities similar to β-W, should have giant SHAs when coupled in heterostructures with Co/Co_40_Fe_40_B_20_/permalloy.

**Fig. 4:**
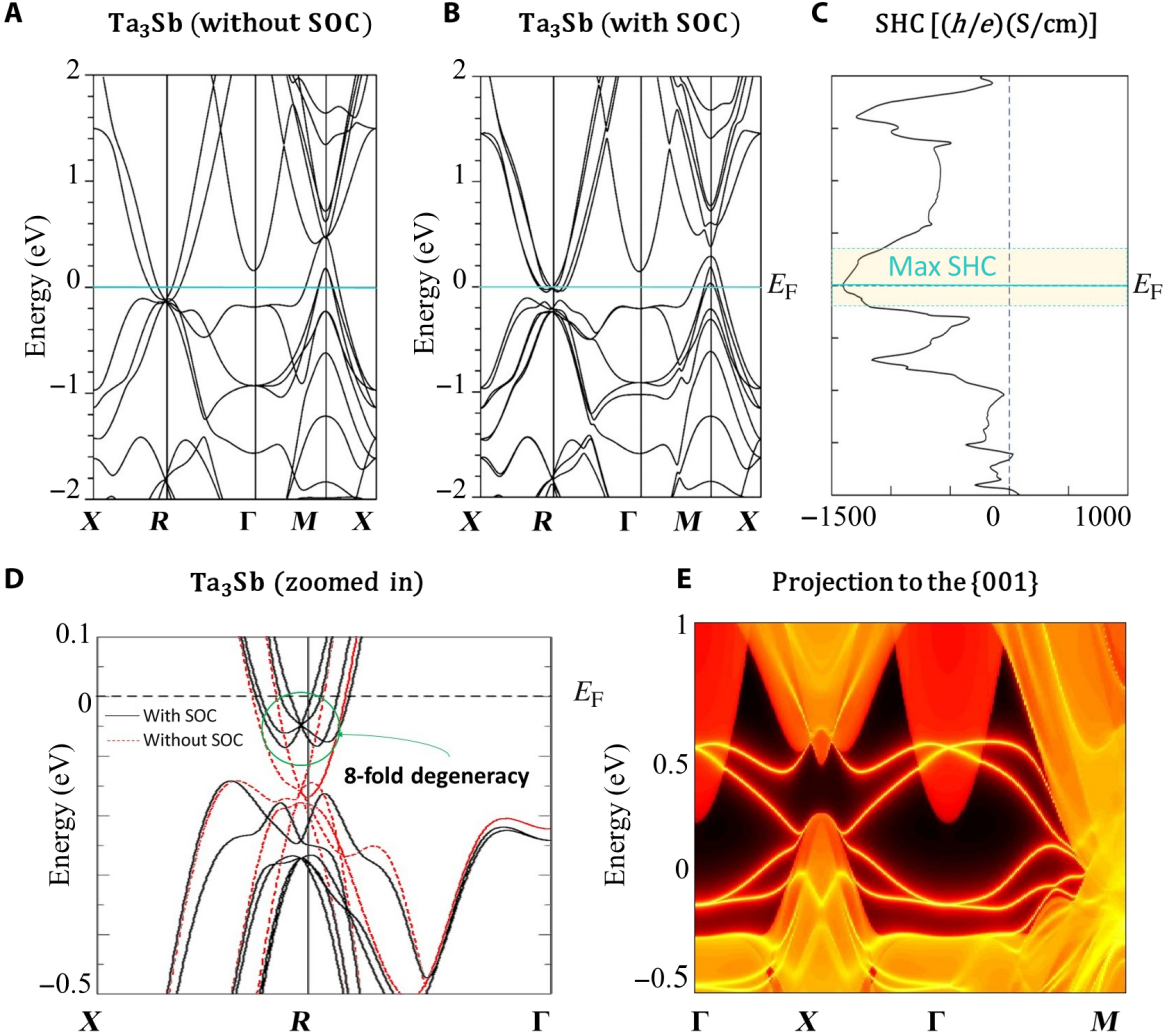
Electronic structure and SHC of Ta_3_Sb. (**A** to **C**) Electronic structure of Ta_3_Sb without and with SOC as well as its SHC versus energy plot. Ta_3_Sb has a peak in its SHC at its *E*_F_. (**D**) Zoomed-in band structure highlighting the eightfold degeneracy near *E*_F_. (**E**) Topological protected surface states (projected to the 001 face) in Ta_3_Sb connecting the conduction and valence bands along *X*-Γ-*M*.

Another A15 compound, Ta_3_Sb, has also an intriguing electronic structure, as shown in Fig. 4 (A and B). The stoichiometric compound has a maximum SHC at *E*_F_ of −1400 $\frac{\hbar }{e}(S/\text{cm})$ (Fig. 4C) as well as an eightfold degenerate Dirac point nearly at *E*_F_ at the *R* point (*41*). When projected to the 001 face, Ta_3_Sb houses topologically nontrivial (fig. S2) surface states, shown as the orange bands in Fig. 4E connecting the conduction bands and valence bands at *X* and *M*. Because this compound is also known to superconduct at 0.7 K (*42*), future experimental studies on both the SHE in this material and the interplay of its topological surface states and superconductivity will be of great interest.

## DISCUSSION

The state of the art in searching for large SHE materials has been limited by a lack of a rational design strategy and a difficult candidate screening process. Materials are experimentally investigated either in a combinatorial, serendipity-driven approach or in a computation-driven approach. The strategy has been to first choose material candidates that are easy to fabricate and contain heavy elements and then calculate electronic structures and Wannier functions before finally calculating the intrinsic SHC using the Kubo formalism. If the final SHC calculation shows a large conductivity, the material is experimentally investigated. This method of screening materials is, however, very time and resource intensive primarily because of the requirement of finding good Wannier functions. Depending on the complexity of the crystal and electronic structures, hundreds of projections need to be attempted before a reasonable one is found and the SHC can be calculated, which is why the search for large SHE materials has been largely dominated by theoretical physicists. However, because we know the descriptor to look for (gapped Dirac or quadractic band crossings near *E*_F_) and given that these crossings can be generated by crystallographic and orbital symmetries, it is possible to markedly cut the screening time of SHC materials by (i) choosing material candidates with high symmetry and heavy elements to generate gapped crossings and (ii) calculating the electronic structures without and with SOC and comparing them to look for the relevant features. Only for candidates with the right features near *E*_F_ does the Wannier projection and SHC need to be calculated. The problem of finding spin Hall materials has been reframed as a pattern recognition problem; high-throughput calculations coupled with artificial intelligence will allow rapid selection. With this simple search strategy, materials scientists, chemists, and experimental physicists who do not have expertise in the details of transport theory can make substantial contributions to the field.

To test this strategy, we expanded our search for large SHE materials to the whole A15 family of materials [many of them are also known to be sputterable for thin-film fabrication (*42*–*46*)]. Electronic structures without and with SOC were calculated for the rest of the family, and only compounds with gap-opened crossings within ±2 eV of *E*_F_ had their SHC calculated. Fitting the pattern, these compounds have maxima in their SHC at energies commensurate with their gapped crossings (see the Supplementary Materials). Several more promising SHE materials were found, and their calculated SHCs at *E*_F_ are listed in Table 1. In addition, alloys of other materials presented here should, like with W_3_Ta, allow *E*_F_ to be adjusted such that the SHC is maximized. Another example of this would be Ti_3−*x*_V_*x*_Pt, where vanadium doping is used to slightly raise *E*_F_ to a peak in its SHC versus energy plot (fig. S15).

**Table 1:** SHCs of selected A15 materials.

**Compounds**	**W_3_Ta**	**Ta_3_Sb**	**Cr_3_Ir**	**Nb_3_Au**	**Ta_3_Au**	**W_3_Re**	**Nb_3_Bi**	**W_3_Si**	**Ta_3_Sn**	**Nb_3_Os**
SHC $\left(\frac{\hbar }{e}(S/\text{cm})\right)$	−2250	−1400	1209	−1060	−870	−780	−670	−640	−620	−460

## CONCLUSION

In summary, we have explained why β-W has a giant SHE, due to its many gapped Dirac crossings resulting in a giant SBC and correspondingly giant intrinsic SHE. We also predicted several more giant SHE compounds, including the low-cost alloy W_3_Ta, which are known to be fabricable in thin-film form via sputtering. From understanding the descriptors in the electronic structure and how it can be caused by symmetry, we also proposed a simple and rapid search strategy for finding materials with large SHE that should enable materials scientists and chemists to contribute to spintronics and make near-future technological impact. Similar to how the search for TIs and Dirac/Weyl materials had an explosion of interest and success due to the approachability of the topic from a variety of different fields, the spin Hall field can also benefit from wide interest. Future work applying the search strategy to other families of compounds such as Heuslers, perovskites, and various intermetallic structural families will show that, like topological materials, large spin Hall materials are actually quite common and that the high-efficiency generation of spin currents is readily achievable in the near future.

## METHODS

Our calculations were performed by using the density functional theory (DFT) with localized atomic orbital basis and the full potential as implemented in the full-potential local-orbital code (*47*). The exchange and correlation energy was considered in the generalized gradient approximation (GGA) level (*48*). The electronic band structures were further confirmed by the calculations from ab initio code of WIEN2k (*49*, *50*). In all the calculations, we adopted the experimentally measured lattice structures. By projecting the Bloch wave functions to the high-symmetry atomic orbital such as Wannier functions, we constructed the tight binding model Hamiltonian. The intrinsic SHCs were calculated from the model Hamiltonian by the Kubo formula approach in the clean limit (*26*, *51*).

$$\begin{array}{c} \sigma_{ij}^{k}=e\hbar \int_{BZ}\frac{d\vec{k}}{{\left(2\pi \right)}^{3}}\sum_{n}f_{n\vec{k}}\Omega_{n,ij}^{k}(\vec{k}),\\ \Omega_{n,ij}^{k}(\vec{k})=-2Im\sum_{n\prime \neq n}\frac{\langle n\vec{k}|J_{i}^{k}|n\prime \vec{k}\rangle \langle n\prime \vec{k}|v_{j}|n\vec{k}\rangle }{{\left(E_{n\vec{k}}-E_{n\prime \vec{k}}\right)}^{2}} \end{array}$$ (4)

The SHC $\sigma_{ij}^{k}$ refers to the spin current ($j_{i}^{s,k}$) flowing along the *i*th direction with the spin polarization along *k*, generated by an electric field (*E*_*j*_) along the *j*th direction, $j_{i}^{s,k}=\sigma_{ij}^{k}E_{j}$. The spin current operator is $J_{i}^{k}=\frac{1}{2}\{\begin{array}{cc} v_{i}, & s_{k} \end{array}\}$, with spin operator *s* and velocity operator $v_{i}=\frac{1}{\hbar }\frac{\partial H}{\partial k_{i}}$ (*i*, *j*, *k* = *x*, *y*, *z*). $|n\vec{k}\rangle$ is the *n*th eigenvector for the Hamiltonian *H* with eigenvalue $E_{n\vec{k}}$, and $f_{n\vec{k}}$ is the Fermi-Dirac distribution for the *n*th band. For convenience, we called $\Omega_{n,ij}^{k}(\vec{k})$ as the SBC as analogy to the ordinary Berry curvature, and the SHCs were computed by the integral of SBC in the BZ with a 500×500×500 *k*-grid.

## Supplementary Material

http://advances.sciencemag.org/cgi/content/full/5/4/eaav8575/DC1

Download PDF

## SUPPLEMENTARY MATERIALS

Supplementary material for this article is available at http://advances.sciencemag.org/cgi/content/full/5/4/eaav8575/DC1 Pt band structure and SHC Ta_3_Sb Z_2_ index A15 band structures and SHCs Fig. S1. The orbital contributions and SHE of Pt. Fig. S2. Topological analysis of Ta3Sb surface states. Fig. S3. Electronic structure without and with SOC included as well as the SHC versus energy plot for W_3_Si. Fig. S4. Electronic structure without and with SOC included as well as the SHC versus energy plot for Nb_3_Os. Fig. S5. Electronic structure without and with SOC included as well as the SHC versus energy plot for Nb_3_Al. Fig. S6. Electronic structure without and with SOC included as well as the SHC versus energy plot for Nb_3_Au. Fig. S7. Electronic structure without and with SOC included as well as the SHC versus energy plot for Nb_3_Bi. Fig. S8. Electronic structure without and with SOC included as well as the SHC versus energy plot for Ta_3_Au. Fig. S9. Electronic structure without and with SOC included as well as the SHC versus energy plot for Ta_3_Ir. Fig. S10. Electronic structure without and with SOC included as well as the SHC versus energy plot for Ta_3_Os. Fig. S11. Electronic structure without and with SOC included as well as the SHC versus energy plot for Ta_3_Sn. Fig. S12. Electronic structure without and with SOC included as well as the SHC versus energy plot for Cr_3_Ir. Fig. S13. Electronic structure without and with SOC included as well as the SHC versus energy plot for Cr_3_Os. Fig. S14. Electronic structure without and with SOC included as well as the SHC versus energy plot for Ti_3_Ir. Fig. S15. Electronic structure without and with SOC included as well as the SHC versus energy plot for Ti_3_Pt. Fig. S16. Electronic structure without and with SOC included as well as the SHC versus energy plot for V_3_Pt. Table S1. SHCs of calculated A15 materials at *E*_F_.
