# Supplementary material for: Giant intrinsic spin Hall effect in W3Ta and other A15 superconductors
Source: Sci Adv. 2019 Apr 5;5(4):eaav8575. doi: 10.1126/sciadv.aav8575 (PMC6450695; doi:10.1126/sciadv.aav8575)
Supplement: http://advances.sciencemag.org/cgi/content/full/5/4/eaav8575/DC1 [file supp_5_4_eaav8575__index.html]

Science Advances | Science Advances

## Supplementary Materials

**This PDF file includes:**

- Pt band structure and SHC
- Ta3Sb Z2 index
- A15 band structures and SHCs
- Fig. S1. The orbital contributions and SHE of Pt.
- Fig. S2. Topological analysis of Ta3Sb surface states.
- Fig. S3. Electronic structure without and with SOC included as well as the SHC versus energy plot for W3Si.
- Fig. S4. Electronic structure without and with SOC included as well as the SHC versus energy plot for Nb3Os.
- Fig. S5. Electronic structure without and with SOC included as well as the SHC versus energy plot for Nb3Al.
- Fig. S6. Electronic structure without and with SOC included as well as the SHC versus energy plot for Nb3Au.
- Fig. S7. Electronic structure without and with SOC included as well as the SHC versus energy plot for Nb3Bi.
- Fig. S8. Electronic structure without and with SOC included as well as the SHC versus energy plot for Ta3Au.
- Fig. S9. Electronic structure without and with SOC included as well as the SHC versus energy plot for Ta3Ir.
- Fig. S10. Electronic structure without and with SOC included as well as the SHC versus energy plot for Ta3Os.
- Fig. S11. Electronic structure without and with SOC included as well as the SHC versus energy plot for Ta3Sn.
- Fig. S12. Electronic structure without and with SOC included as well as the SHC versus energy plot for Cr3Ir.
- Fig. S13. Electronic structure without and with SOC included as well as the SHC versus energy plot for Cr3Os.
- Fig. S14. Electronic structure without and with SOC included as well as the SHC versus energy plot for Ti3Ir.
- Fig. S15. Electronic structure without and with SOC included as well as the SHC versus energy plot for Ti3Pt.
- Fig. S16. Electronic structure without and with SOC included as well as the SHC versus energy plot for V3Pt.
- Table S1. SHCs of calculated A15 materials at *E*F.

Download PDF

**Files in this Data Supplement:**

- Adobe PDF - aav8575\_SM.pdf
