# Supplementary material for: Giant intrinsic spin Hall effect in W3Ta and other A15 superconductors
Source: Sci Adv. 2019 Apr 5;5(4):eaav8575. doi: 10.1126/sciadv.aav8575 (PMC6450695; doi:10.1126/sciadv.aav8575)
Supplement: Download PDF [file aav8575_SM.pdf]

Science Advances
AAAS

advances.sciencemag.org/cgi/content/full/5/4/eaav8575/DC1

Supplementary Materials for

Giant intrinsic spin Hall effect in W_3Ta and other A15 superconductors

E. Derunova, Y. Sun, C. Felser, S. S. P. Parkin, B. Yan, M. N. Ali*

*Corresponding author. Email: maz@berkeley.edu

Published 5 April 2019, *Sci. Adv.* **5**, eaav8575 (2019)
DOI: 10.1126/sciadv.aav8575

This PDF file includes:

Pt band structure and SHC
Ta_3Sb Z_2 index
A15 band structures and SHCs
Fig. S1. The orbital contributions and SHE of Pt.
Fig. S2. Topological analysis of Ta3Sb surface states.
Fig. S3. Electronic structure without and with SOC included as well as the SHC versus energy plot for W_3Si.
Fig. S4. Electronic structure without and with SOC included as well as the SHC versus energy plot for Nb_3Os.
Fig. S5. Electronic structure without and with SOC included as well as the SHC versus energy plot for Nb_3Al.
Fig. S6. Electronic structure without and with SOC included as well as the SHC versus energy plot for Nb_3Au.
Fig. S7. Electronic structure without and with SOC included as well as the SHC versus energy plot for Nb_3Bi.
Fig. S8. Electronic structure without and with SOC included as well as the SHC versus energy plot for Ta_3Au.
Fig. S9. Electronic structure without and with SOC included as well as the SHC versus energy plot for Ta_3Ir.
Fig. S10. Electronic structure without and with SOC included as well as the SHC versus energy plot for Ta_3Os.
Fig. S11. Electronic structure without and with SOC included as well as the SHC versus energy plot for Ta_3Sn.
Fig. S12. Electronic structure without and with SOC included as well as the SHC versus energy plot for Cr_3Ir.
Fig. S13. Electronic structure without and with SOC included as well as the SHC versus energy plot for Cr_3Os.
Fig. S14. Electronic structure without and with SOC included as well as the SHC versus energy plot for Ti_3Ir.

Fig. S15. Electronic structure without and with SOC included as well as the SHC versus energy plot for Ti_3Pt.
Fig. S16. Electronic structure without and with SOC included as well as the SHC versus energy plot for V_3Pt.
Table S1. SHCs of calculated A15 materials at E_F.

Pt band structure and SHC

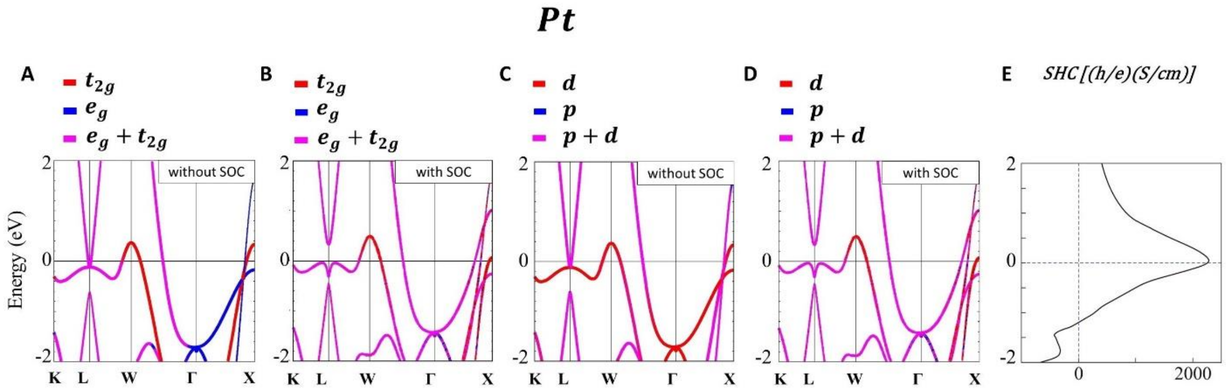

Fig. S1. The orbital contributions and SHE of Pt. Panels (**A**) and (**B**) show the orbital contributions to the electronic band structure of platinum grouped by symmetry. Panels (**C**) and (**D**) show contributions grouped by atomic orbitals. (**E**) The calculated spin Hall conductivity. Gap opened crossings are seen at the L-point and along the Γ - X line.

Ta_3Sb Z_2 index

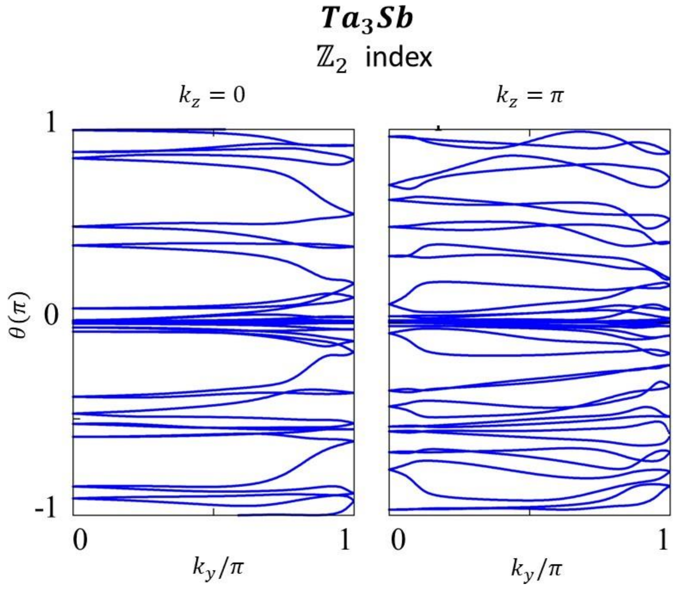

Fig. S2. Topological analysis of Ta3Sb surface states. Index plot for the Ta_3Sb surface states, showing their non-trivial topological nature.

A15 band structures and SHCs

Table S1. SHCs of calculated A15 materials at E_F.

Compounds	W_3Ta	W_3W	Ta_3Sb	Cr_3Ir	Nb_3Au	Ta_3Au	W_3Re	Ta_3Ta	Nb_3Bi	W_3Si
SHC ($\frac{\hbar}{e}$(S/cm))	-2250	-1900	-1400	1209	-1060	-870	-780	-720	-670	-640

Compounds	Ta_3Sn	Nb_3Os	Nb_3Al	V_3Pt	Ti_3Pt	Ta_3Os	Ta_3Ir	Ti_3Ir	Cr_3Os
SHC ($\frac{\hbar}{e}$(S/cm))	-620	-460	-440	-440	330	-230	-143	56	-40

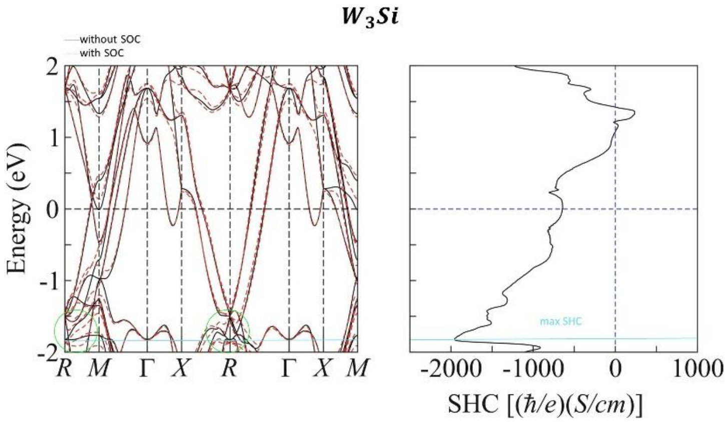

Fig. S3. Electronic structure without and with SOC included as well as the SHC versus energy plot for W_3Si.

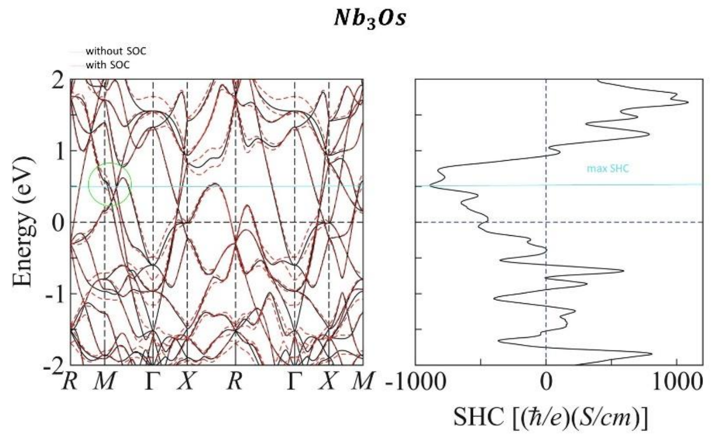

Fig. S4. Electronic structure without and with SOC included as well as the SHC versus energy plot for Nb_3Os.

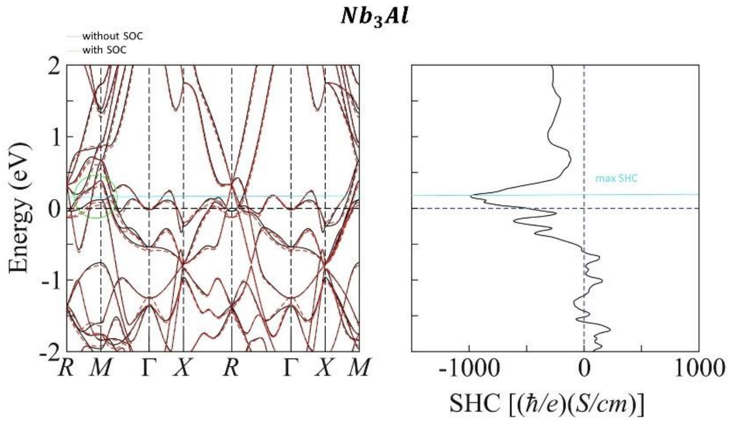

Fig. S5. Electronic structure without and with SOC included as well as the SHC versus energy plot for Nb_3Al.

Nb_3Au

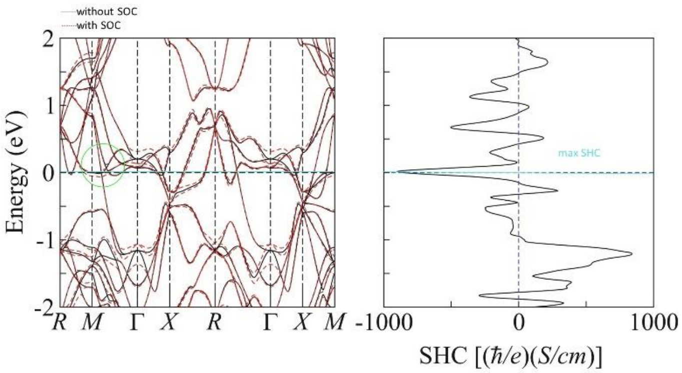

Fig. S6. Electronic structure without and with SOC included as well as the SHC versus energy plot for Nb_3Au.

Nb_3Bi

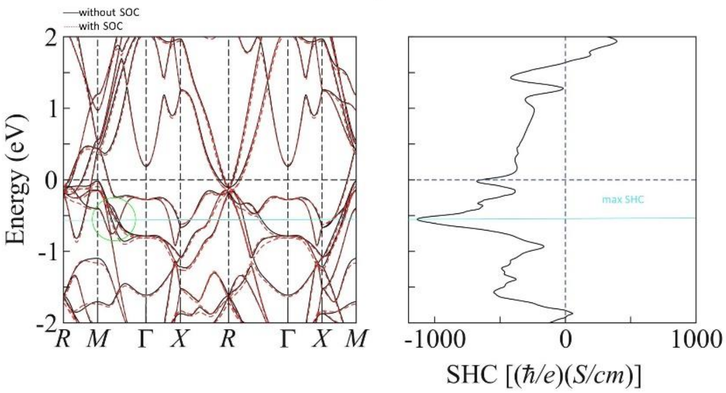

Fig. S7. Electronic structure without and with SOC included as well as the SHC versus energy plot for Nb_3Bi.

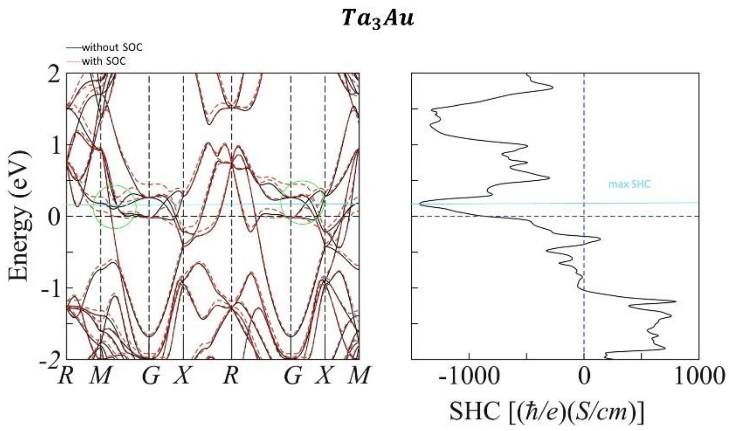

Fig. S8. Electronic structure without and with SOC included as well as the SHC versus energy plot for Ta_3Au.

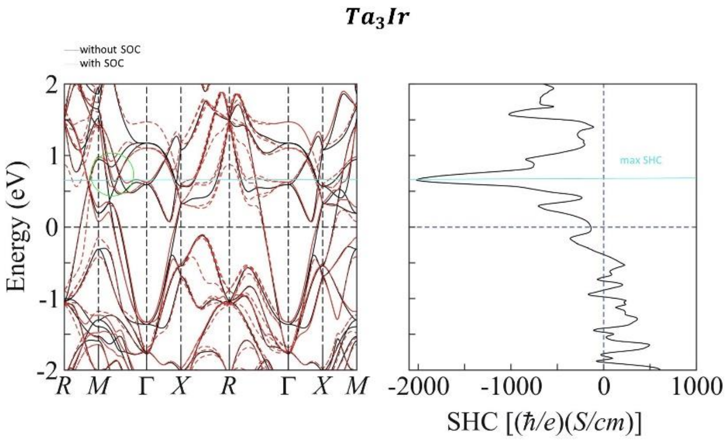

Fig. S9. Electronic structure without and with SOC included as well as the SHC versus energy plot for Ta_3Ir.

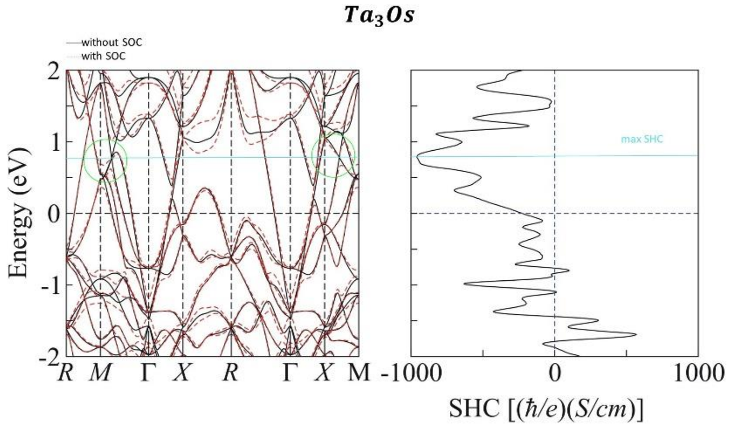

Fig. S10. Electronic structure without and with SOC included as well as the SHC versus energy plot for Ta_3Os.

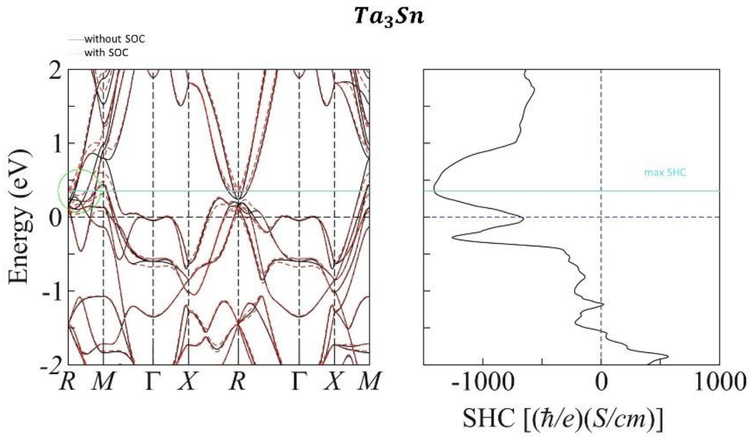

Fig. S11. Electronic structure without and with SOC included as well as the SHC versus energy plot for Ta_3Sn.

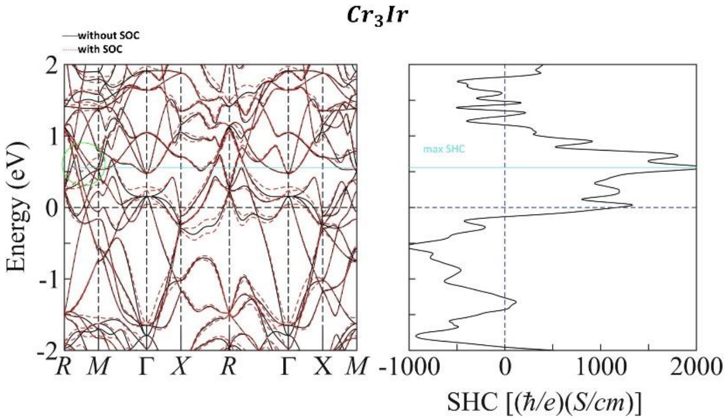

Fig. S12. Electronic structure without and with SOC included as well as the SHC versus energy plot for Cr_3Ir.

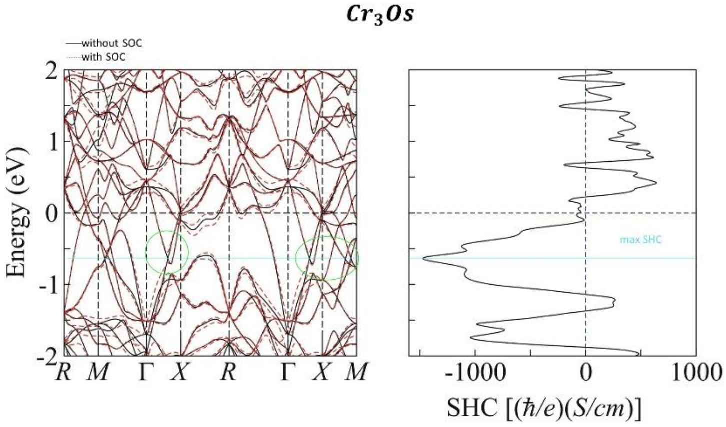

Fig. S13. Electronic structure without and with SOC included as well as the SHC versus energy plot for Cr_3Os.

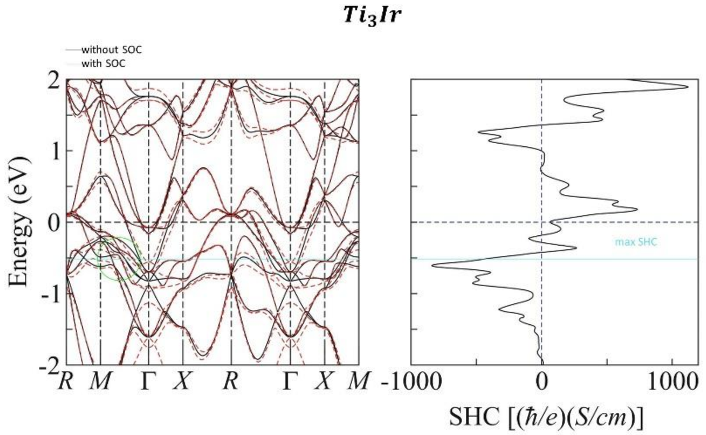

Fig. S14. Electronic structure without and with SOC included as well as the SHC versus energy plot for Ti_3Ir.

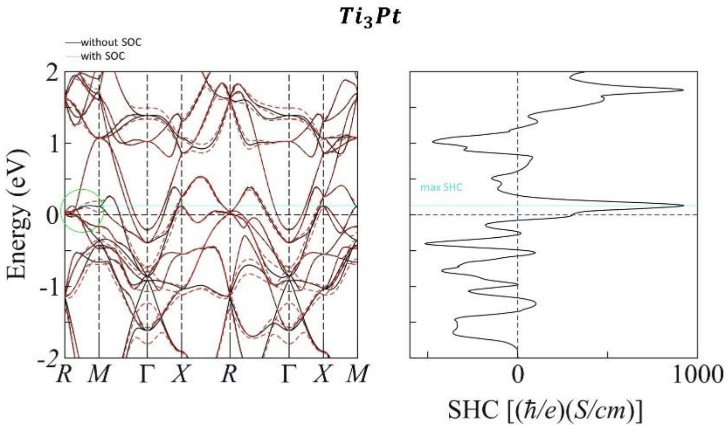

Fig. S15. Electronic structure without and with SOC included as well as the SHC versus energy plot for Ti_3Pt.

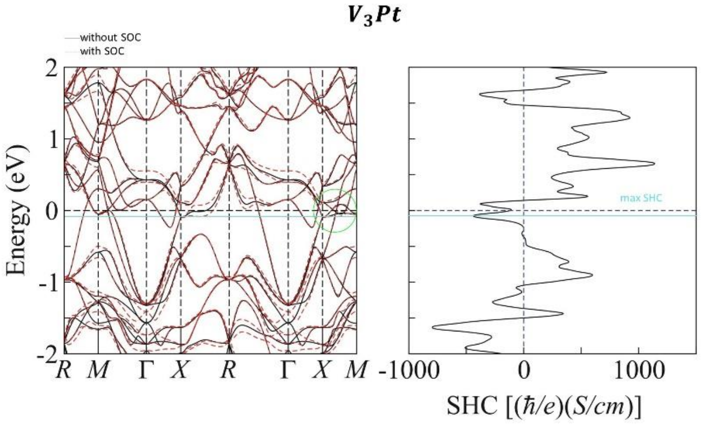

Fig. S16. Electronic structure without and with SOC included as well as the SHC versus energy plot for V_3Pt.
